# Magnitude and factors for method discontinuation and switching among long acting reversible contraceptive users in health facilities of Southern Ethiopia

**DOI:** 10.1186/s12978-022-01357-2

**Published:** 2022-02-19

**Authors:** Tessema Bereku, Yitagesu Habtu, Bereket Abreham, Menen Ayele, Melesech Eliso

**Affiliations:** 1 Department of Midwifery, Hossana College of Health Sciences, P.O. Box: 159, Hossana, Ethiopia; 2 Department of Public Health, Hossana College of Health Science, Hossana, Ethiopia; 3 Department of Health Extension Services, Hossana College of Health Sciences, Hossana, Ethiopia; 4 Department of Clinical Nurses, Hossana College of Health Sciences, Hossana, Ethiopia; 5 Department of Midwifery, Hossana College of Health Sciences, Hossana, Ethiopia; 6 Present Address: College of Health Sciences, School of Public Health, Addis Ababa University, Addis Ababa, Ethiopia

**Keywords:** LARCs, Discontinuation, Switching, Primiparous

## Abstract

**Background:**

Long Acting Reversible Contraceptives (LARCs) are contraceptives that prevent unplanned pregnancy in a more safer and effective way than other modern short acting methods. However, method discontinuation and switching are still challenges for utilization of LARCs in resource limited countries for several reasons. Thus, the aim of this study was to determine magnitude and factors for method discontinuation and switching among LARCs users in health facilities of Southern Ethiopia.

**Methods:**

A Facility based record review was used to collect data from May to June 2019. Three hospitals were randomly selected from five hospitals found in southern Ethiopia. A total of 1050 records were included in the study from long acting family planning registers between 2018 and 2019. Data were entered to Epi-info 3.5.4 and exported to SPSS for windows version 20 for analysis. A descriptive statistics was performed to describe factors and reasons for LARCs discontinuation and switching off. Logistic regression technique with a 95% confidence level was used to determine the association between factors and magnitude of method discontinuation and switching.

**Results:**

Of the 1050, 69.8% of women discontinued long acting reversible family planning method before the recommended duration of use and 30.2% of them switched from long acting family planning methods to any other modern contraceptive methods. Women who shifted from any LARCs to short-acting family planning methods accounted for 38.8% of those who shifted to any other modern methods. Desire to get pregnant and method specific side effect were most common reasons for both method discontinuation and switching. Women with only one child were 1.61 times more likely to discontinue than women who had greater than five number of children.

**Conclusion:**

Discontinuation and switching of long acting reversible family planning method was high. Primiparous women were more likely to discontinue use of long acting reversible family planning methods. Re-evaluating family planning services focusing on effective counseling about side effects of LARCs methods is required. Training should also be given for family planning providers including community healthcare workers.

**Supplementary Information:**

The online version contains supplementary material available at 10.1186/s12978-022-01357-2.

## Plain language summary

Ethiopia has a substantially greater percentage of unintended pregnancy than other developing and developed countries. Long-acting reversible contraception (LARC) has a potential to address a huge and growing unmet need in resource limited countries like Ethiopia, such as reducing unwanted births and abortion rates.

As part of this global approach to fertility management, Ethiopia has been implementing various strategies like an on-going task shifting and task sharing with the implanon scale up program, and IUD revitalization program starting from the community health services to higher level healthcare systems to increase access and utilization of long acting reversible family planning methods. However, discontinuing LARCs before the recommended duration use and switching to short-acting contraceptives have proven difficulty in Ethiopia. This may have led to low utilization rate of LARCs in Ethiopian contexts including the study area, when compared to the national target. Very low coverage of use of LARC among unmet need has shown by many studies in various corners of the country. Thus, determining the magnitude and factors of LARC discontinuing and switching is still a pressing need to establish additional strategies used to improve consistent use of LARC for the recommended duration and expand access to LARC for better planning of births.

In this study we assessed magnitude and factors of LARC discontinuation and switching from a relatively huge number of records extracted from systematically selected health facilities in Southern Ethiopia. Extracted data from registers of long acting reversible family planning methods were analysed using descriptive statistics and logistic regression. Accordingly, we found significant proportion women discontinued long acting reversible family planning methods before the recommended duration of use as well as switched from long acting reversible family planning methods to other modern short-acting methods due to various reasons. Only a number of children women had is associated with discontinuation and no single factor was associated with method switching in Southern Ethiopia. Family planning services should be re-evaluated with a focus on effective counseling on the side effects of LARCs approaches, and training for family planning providers, particularly community healthcare workers, should be provided.

**Supplementary Information:**

The online version contains supplementary material available at 10.1186/s12978-022-01357-2.

## Background

Family planning is one of the most cost effective health promotion programs reducing maternal and child mortality by roughly 30% and 10%, respectively [1]. Despite significant reduction of maternal and child mortality due to fertility related causes from 1990, the burden of both maternal and child mortality high in low resource setting countries Approximately 295,000 maternal deaths occurred globally in 2017 and 94% of which was from low resource countries. Sub-Saharan Africa and South Asia accounted for 86% (254,000) of global maternal deaths in 2017 [2]. Similarly, 5.4 million children died in 2017 globally and 89% of them are from low and middle income countries. Fortunately, the vast majority of maternal and child deaths can be prevented with proven interventions including effective birth control using modern contraceptions mainly LARCs [3].

Worldwide contraceptive utilization has increased during the last 10 years [4]. Unfortunately, many other regions of the developing world including Africa still have a high unmet need for family planning. In sub-Saharan Africa, an estimated 25% of women and couples who want to space or limit their births are not using any form of contraception. Because more than half of Africa’s population is under the age of 25, unmet need for family planning is likely to rise as these individuals enter their reproductive years [5].

Long-acting reversible contraceptives (LARCs) are contraceptive methods that prevent unplanned pregnancy for a long period of time without the need to any further responsibility by the user. They include the Intrauterine Contraceptive Devices (IUCD), Implants and Jadelle [6, 7]. Such modern contraceptives are more effective and cost-effective methods that play a role in reaching the national and international birth control goals.

The government of Ethiopia, national fertility rate of 4.6 children per women with a 2.7% per year population growth rate [8] has targeted 55% contraceptive prevalence rate by the year 2020. Of which, 35% is targeted for LARCs [9] implying that much needs to be done to catalyze the uptake of these methods. Despite this target, the utilization coverage of LARCs remains small sometimes missed component of many national reproductive health and family planning programs [5]. In light of this, the country has implemented different strategies such as task shifting to enhance the utilization coverage of LARCs at the primary healthcare level in all regions, including the southern region.

Method discontinuation and switch off are challenges for underutilization of most effective modern contraceptives, LARCs in limited resource settings for several reasons [10, 11]. Method discontinuation in this study refers to stopping use of specific LARCs within the recommended duration and discontinuing the previous method and switching to another LARCs [12]. Desire to conceive, unexpected conception, and side effect or health concern were the most prevalent reasons reported for discontinuing contraceptive methods, regardless of the method currently in use [11]. Method shift (switch off) from LARCs [13] to any other short-acting methods mainly due to poor competency of providers in counseling, providers’ gender, providers’ position and sector, provider myths is one of the reasons for inconsistent use of LARCs [13].

In low income countries including Ethiopia, the proportion of family planning users who switch off from reversible long acting family planning methods to less effective methods reaches up to 40.4% [13]. According to the 2016 Ethiopian Demographic Health Survey (EDHS), percentage of long acting reversible contraceptive method episodes discontinued within 12 months were IUCD (13%) followed by implants (11%) among women age 15–49 [14].

Southern nation's nationalities and people’s regional health bureau of Ethiopia set a strategic plan to achieve the coverage of long acting reversible family planning users from 24 to 60% according to the Health Sector Transformation Plan (HSTP) in 2020 [9]. However, the utilization coverage of the long acting reversible family planning is significantly lower than the expected coverage due to various reasons. Discontinuation from using and switching from long acting reversible family planning methods to short acting methods might contribute to low utilization of long acting reversible family planning methods. The reasons for discontinuation and switching might be highly contextual depending on cultural variations and time elapse. Method discontinuation and switching from long acting family planning to short acting modern or traditional methods leads to the risk of unplanned pregnancy [15] that may expose for unsafe abortion [16] and Sexually transmitted infections (STIs)/ Human immunodeficiency virus (HIV) infection [17]. However, information regarding methods discontinuation from using long acting reversible family planning methods and shift from highly effective method to less effective methods is largely unknown in the study area. Therefore, the aim of this study was to determine the magnitude of discontinuation and switching, and reasons for method discontinuation and switching off long acting reversible contraceptives.

## Methods

### Study setting

The study was done in three randomely selected hospitals found in central Southern Ethiopia, Hadiya Zone. There were five hospitals providing family planning services including LARCs during data collection period. The hospitals include Wachamo University Nigist Eleni Mohamed Memorial referral Hospital (WUNEMMRH), Shone Primary Hospital, Gimbichu Primary Hospital, Homecho primary hospital and Bonesha primary hospital. All hospitals have been providing family planning services since family planning program and strategies began to be implemented. According to the national health information system of the country, all healthcare facilities are expected to register LARCs users using a separate standard registration book since 2018. IUCD, implants (Implanon, and Jadellele) are available LARCs in all family planning services in Southern Ethiopia including the study area. There were more than 1200 long acting family planning users every year in the catchment area according to 2018 report of the Zone. The coverage of long acting reversible family planning service utilization was reported to be low; ranging from 15 to 20% among the eligible women in 2018 according to reports of Zonal Health Bureau [18].

### Study design and period

A facility based record review was used to assess magnitude, reasons and factors of discontinuation and switching among long acting reversible family planning (LARCs) users in public health facilities in Hadiya Zone in Central Southern Ethiopia from May to June 2019.

### Study participants and sampling

Data are extracted from records of long acting reversible family planning users who revisit the hospital family planning unit. A total of 1050 records in long acting family planning registers from 2018 to 2019 were included in the study from three randomly selected hospitals found in Hadiya Zone. The records from 2018 to 2019 were used because of healthcare facilities had started using a standard LARCs registration book since 2018–2019, the end of the study period. For the record review, three hospitals were chosen at random from a list of five hospitals as sampling frame in zonal health bureaus, assuming that the LARCs users have little variation in contextual factors with regard to socio-demographic and other characteristics. In addition, all hospitals have been fully providing long acting reversible family planning services and started to use a standard LARCs registration book.

### Study variables

The dependent variables of the study were discontinuation of long acting reversible family planning methods and switching off from long acting reversible family planning to any other modern methods. Method discontinuation for this study refers to when the woman stop using a previously chosen specific LARC within the recommended duration of use. Method switching for this study refers to the case where a woman made shifts from currently using a specific LARCs to other specific LARC, or other specific modern contraceptives. LARCs for this study applies to available modern contraceptives across all family planning services of healthcare facilities in the study area such as implants (implanon and Jadellele) and IUCD.

Whereas, the independent variables include socio-demographic characteristics of users, types of family planning methods previously used, and HIV testing status. Socio-demographic variables include age, marital status, education, place of residence, parity, and gravidity.

### Data collection and measurement

A checklist with similar data items of the standard LARCs registries and women card was prepared to collect the relevant information about the users who revisit family planning unit. For more clarity, see the English version checklist (Additional file 1: Annex 1). Socio-demographic and related data that were not available in the standard LARCs registries were extracted from the women cards. Long acting reversible family planning users who came to discontinue the LARC which has been in use and came to switch to other methods for any reasons were extracted to determine the magnitude of discontinuation and switching.

Records available before 2018 were excluded because all five hospitals were not equally using a standard national LARCs registries. I Similarly, incomplete records were excluded from analysis. The data collectors and supervisors were trained how to extract the data using the checklists that had been prepared. The supervisors monitored the data collection process regularly on a daily basis. Daily checkup was made to ensure completeness and consistency of the data in the checklists.

### Data processing and analysis

Data recorded on the checklists were entered to Epi-info 3.5.4 after checking consistency and completeness. Then, the data were exported to SPSS for windows version 20 for performing statistical analysis.

A descriptive statistics was performed to describe factors and reasons for LARC discontinuation and switching off. The variables were described using a variety of frequency tables, charts, and summary measures. Proportion was used to determine magnitude of both outcome variables (discontinuation and Switching off LARCs) separately.

Logistic regression with standard entry method was used to determine the association between socio-demographic variables and magnitude of discontinuation; socio-demographic variables and switching off LARCs. We computed odds ratio with 95% confidence interval to show the strength of the association between independent variables and dependent variables (magnitude of discontinuation and switching off). Bivariate logistic regression with standard method was used to see the crude association between independent variables and magnitude of discontinuation. We used a similar step to determine the crude association between independent variables and magnitude of switching off LARCs. Despite we planned to take variables having statistically significant results (P-value < 0.05) to the multiple logistic regression, there was only one eligible variable (number of children the women have) with both magnitude of discontinuation so that the result obtained in bivariate logistic regression model was taken as independent factor for discontinuation of LARCs. We used a similar step in the case of switching off LARCs however there was no single variable associated with switching off LARCs in a bivariate logistic regression.

## Results

### Socio-demographic characteristics of women

A total of 1050 women were included in the analysis. The median age of the women was about 28 years and the minimum age was 15 while the maximum age was 45. Majority of women were younger than 35 years old and while only 2.2% were in the later adolescent age category. Nearly one in ten women had not received formal education whereas more than half of the women had attained primary and secondary level education. The number of children who had ever born from the women was ranged from 0 to 7 while the number of times that a woman had been pregnant ranged from 0 to 8. A vast majority of the women, 96.1%, had married. More than half of the women, 55.8%, resided at rural area. The distribution of socio-demographic characteristics is depicted in Table 1.

**Table 1:** Distribution of socio-demographic characteristics of women of long acting family planning users, Hadiya Zone, Ethiopia, 2019

Variables	Frequency	Percent
Women’s age at times of removal of LARCs		
15–24	222	21.1
25–34	680	64.8
35 or More	148	14.1
Education		
Illiterate	102	9.7
Primary education	281	26.8
Second education	294	28.0
College or above	373	35.5
Place of residence		
Rural	586	55.8
Urban	464	44.2
Marital status		
Single	12	1.1
Married	1009	96.1
Divorced	17	1.6
Widowed	12	1.1
Number of living children		
No child	25	2.4
One child	225	21.4
Two to four child	577	55.0
Five or more child	223	21.2
Total pregnancy		
No pregnancy	4	0.4
One pregnancy	383	16.2
Two to four pregnancy	477	51.8
Five or more pregnancy	186	31.6

### Discontinuation and switching of LARCs methods

Of the 1050 users of LARCs, the methods chosen were implanon, IUCD and Jadelle in the period before removal. Implanon was the most common long acting reversible family planning method used by 871 (83%) women followed by intrauterine contraceptive device (IUCD) 91 (8.7%), and Jadelle 88 (8.4%).

Seven hundred and thirty three (69.8%) of women discontinued the specific LARC within 1 year of insertion without shifting to any other modern contraception. Whereas 317 (30.2%) of women switched from long acting family planning methods to any other modern contraceptive methods. Of the 317 women, 123 (38.8%) of them were switched from the long acting reversible contraceptives to short acting modern family planning methods whereas 194 (61.2%) of them were shifted from one specific LARC to another LARC.

The mean duration of use for all long acting reversible family planning methods was 19.3 ± 15.2 months. The mean durations of use for Implanon, IUCD and Jadelle were 19.3 ± 12.2, 24.4 ± 26.8 and 31.8 ± 22.2 months, respectively. Table 2 presents all-method and method-specific duration of use among women who discontinued a specific method. One hundred twenty two (16.6%) of women discontinued for all methods after 6 months protection of unintended pregnancy and increased to 40.4% after more than 24 months protection of unintended pregnancy. Implanon had the lowest 6 month discontinuation rate (15.4%) followed by Jadelle (16.9%). More than a quarter of women discontinued IUCD (28.8%) within 6 months duration of use. The highest discontinuation rate is recorded for Jadelle (55.9%) followed by IUCD (44.1%) after 2 years of use.

**Table 2:** Percentage of women who discontinued a method at 6, 12, 18, 24, and more than 24 months duration of use of LARCs, by method, Hadiya Zone, Ethiopia 2019

Method	6 months	12 months	18 months	24 months	More than 24 months	Total
	Number (%)	Number (%)	Number (%)	Number (%)	Number (%)	Number (%)
Implanon	95 (15.4%)	115 (18.7%)	101 (16.4%)	67 (10.9%)	237 (38.5%)	615 (100.0%)
Jadelle	10 (16.9%)	5 (8.5%)	9 (15.3%)	2 (3.4%)	33 (55.9%)	59 (100.0%)
IUCD	17 (28.8%)	10 (16.9%)	3 (5.1%)	3 (5.1%)	26 (44.1%)	59 (100.0%)
Total	122 (16.6%)	130 (17.7%)	113 (15.4%)	72 (9.8%)	296 (40.4%)	733 (100.0%)

Among those women who switched from the specific LARC, 256 (80.8%) switched from implanon to other modern contraceptive methods. Of which, 107 (41.8%) switched from implanon to other short acting methods (male condom, pills, injectables) and 149 (58.2%) switched from implanon to other long acting reversible family planning methods (IUCD and Jadelle). Thirty two (10.1%) switched from IUCD to other methods and 29 (9.2%) switched from Jadelle to other methods (Table 3).

**Table 3:** Previously used long acting reversible family planning methods and post removal contraceptive family planning methods, Hadiya Zone, Ethiopia 2019

Method	Male condom	Pills	Injectables	IUCD	Implant	No contraception	Total
	Number (%)	Number (%)	Number (%)	Number (%)	Number (%)	Number (%)	Number (%)
Implanon	10 (1.0%)	48 (4.6%)	49 (4.7%)	96 (9.1%)	53 (5.0%)	615 (58.6%)	871 (83.0%)
Jadelle	1 (0.1%)	0 (0%)	2 (0.2%)	9 (0.9%)	17 (1.6%)	59 (5.6%)	88 (8.4%)
IUCD	0 (0.0%)	6 (0.6%)	7 (0.7)%	3 (0.3%)	16 (1.5%)	59 (5.6%)	91 (8.7%)
Total	11 (1.0%)	54 (5.1%)	58 (5.5%)	108 (10.3%)	86 (8.2%)	733 (69.8%)	1050 (100.0%)

The women’s initial long-acting reversible family planning method and destination method is depicted in Table 3. The majority of women who were using implanon 615 (58.6%) stopped from using any modern contraceptive methods after removal. The three most modern contraceptives preferred by women after removal of Implanon were IUCD 96 (9.1%), implant (Jadelle or implanon) 53 (5.0%), and injectables 49 (4.7%). Out of women who were using Jadelle, 59 (5.6%) of them stopped from using the method. Whereas, 17 (1.6%) of them shifted to implant (implanon or Jadelle).

### Reasons for discontinuation and switching off LARCs

**Fig. 1:**
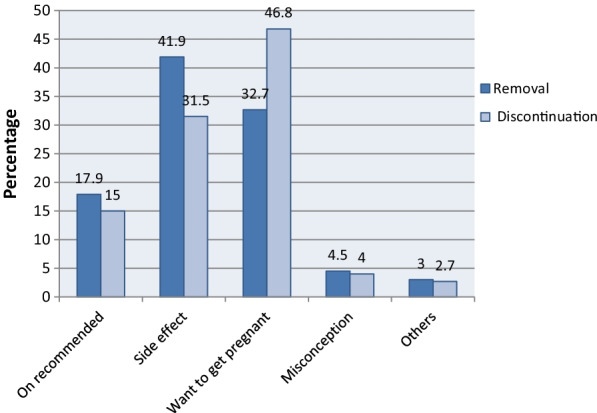
Reasons for removal and discontinuation of long acting reversible family planning methods, Hadiya Zone, Ethiopia

Of the total women who were using specific LARC, 440 (41.9%) of them sought removal services due to method specific side effects, followed by desire to get pregnant 343 (32.7%). The two most common reasons for discontinuing a method were desire to become pregnant 343 (46.8%) followed by method specific side effects 231 (31.5%) among women who discontinued using it. Of women who discontinued the specific LARC, 390 (53.2%) of them discontinued with reasons other than desire to become pregnant. Only 188 (17.9%) of women sought removal services, and 110 (15%) of those who discontinued specific LARC waited for the due date (the time when the effective date of the method was over). A few other women reported various reasons such as husband disapproval, marital dissolution, and so on (Fig. 1).

The two most important reasons given by the women who came for removal of implanon were side effects 359 (34.2%) followed by desire to get pregnant 289 (27.5%). Most common reasons reported by the women who were using Jadelle were side effect, 34 (3.2%) followed by at due date of use 26 (2.5%). Similarly, the commonest reasons provided by the IUCD users for removal were the side effects felt followed by desire to get pregnant (Table 4).

**Table 4:** Reasons provided by the women who came to remove long acting reversible family methods by type of methods, Hadiya Zone, Ethiopia 2019

Reasons	Implanon	Jadelle	IUCD	Total
	Number (%)	Number (%)	Number (%)	Number (%)
On recommended	158 (15.0%)	26 (2.5%)	4 (0.4%)	188 (17.9%)
Side effect	359 (34.2%)	34 (3.2%)	47 (4.5%)	440 (41.9%)
Want to get pregnant	289 (27.5%)	24 (2.3%)	30 (2.9%)	343 (32.7%)
Misconception	37 (3.5%)	1 (0.1%)	9 (0.9%)	47 (4.5%)
Others	28 (2.7%)	3 (0.3%)	1 (0.1%)	32 (3.0%)
Total	871 (83.0%)	88 (8.4%)	91 (8.7%)	1050 (100.0%)

**Fig. 2:**
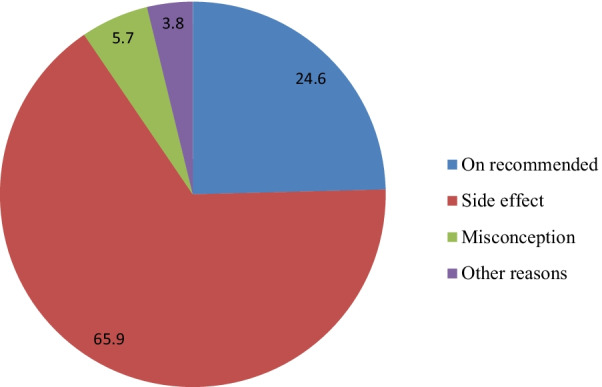
Reasons for switching from long acting reversible family planning to other family planning methods

As shown on Fig. 2, among those women who switched from long acting reversible family planning to any method, the common reasons were side effect 209 (65.9%) followed by due date of use of the method 78 (24.6%).

### Factors affecting discontinuation and switching off LARCs

Despite we planned to take variables having a P-Value less than 0.05 to the multiple logistic regression analysis, only number of children was significantly associated factors with discontinuation of long acting family planning methods in the bivariate analysis. Those women who had one living children were 1.61 times more likely to discontinue than women who had greater than five number of pregnancy [AOR: 1.61, 95% CI: 1.61(1.07, 2.42)] (Table 5). In bivariate logistic regression, there was no single factor associated with switching off LARCs; hence no factors were eligible for further multiple logistic regression analysis. Therefore, there was no statistically significant association between factors and switching off LARCs.

**Table 5:** Factors associated with discontinuation of long acting reversible family planning in Hadiya zone, Ethiopia, June 2019

Variable	Variable category	Frequency	Crude OR 95% CI	P value	Adjusted OR 95% CI	P value
Women’s age at times of discontinuation	15–24	23	1			
	25–34	199	1.0 (0.72, 1.40)	0.997		
	35 or more	442	0.74 (0.48, 1.16)	0.187		
Place of residence	Rural	586	0.84 (0.63, 1.09)	0.173		
	Urban	464	1			
Education of respondent*	Illiterate	102	0.96 (0.60, 1.53)	0.849		
	Primary education	281	1.05 (0.75, 0.47)	0.759		
	Second education	294	1.18 (0.85, 1.65)	0.330		
	College or above	373	1			
Marital status	Single	12	1			
	Married	1009	1.66 (0.52, 5.29)	0.388		
	Divorced	17	1.31 (0.29, 5.98)	0.728		
	Widowed	12	2.14 (0.38, 12.20)	0.390		
Number of living children	No child	25	0.71 (0.31, 1.64)	0.426	0.71 (0.31, 1.64)	0.426
	One child	225	1.61 (1.07, 2.42)	0.021	1.61 (1.07, 2.42)	0.021
	Two to four child	577	1.36 (0.98, 1.89)	0.064	1.36 (0.98, 1.89)	0.064
	Five or more child	223	1		1	
Total number of pregnancy	No pregnancy	4	1.57 (0.16, 15.43)	0.697		
	One pregnancy	383	1.37 (0.94, 1.99)	0.100		
	Two to four pregnancy	477	1.19 (0.83, 1.70)	0.345		
	Five or more pregnancy	186	1			
Types of LARCs	Implanon	871	1			
	Jadelle	88	0.85 (0.53, 1.35)	0.486		
	IUCD	91	0.77 (0.49, 1.21)	0.254		
Duration in months	6 or less	173	0.95 (0.65, 1.41)	0.812		
	6.1–12.00	195	0.80 (0.55, 1.15)	0.225		
	12.1–18.0	168	0.82 (0.56, 1.21)	0.311		
	18.1–24	100	1.03 (0.63, 1.67)	0.920		
	24.1 or more	414	1			

Eligible variables for multivariate analysis (P < 0.05), but no variables were elligible for multiple logistic regression

## Discussion

Despite years had been counted since we had started implementing birth control through family planning methods, there is lower rate of utilization and early discontinuation and switching of long acting family planning methods that may expose women for unintended pregnancy. The present study determined the magnitude of early discontinuation and switching of common long acting modern family planning methods, reasons for discontinuation and factors associated with early discontinuation.

This study found that about seven in ten women discontinued any long acting reversible family planning method. This is by far higher than the findings from Honduras, where 41% of women discontinued using their first choice of LARC within 1-year follow up [19] and a Kenyan study, which found that the 12-month contraceptive discontinuation rate for all the methods was 30.5% and IUCD (6.4%) followed by Implants (8%), in 2014 [20]. In addition, it was higher than from the finding of the Ethiopian national health survey which indicated overall discontinuation rate of 35% for all the methods and method specific 1 year discontinuation of IUCD (13%) followed by Implants (11%) [14]. In fact, the findings of the EDHS may not be incomparable with this study due to the fact that the design of EDHS is population based whereas this study is facility based and among high risk group for discontinuation [14] 

] Furthermore, the finding of the study was higher than the study from South East Ethiopia specifically for implanon [7] where 18.7% of women removed implanon within first year of use and (38.5%) of women removed implanon after the second year of use. However, higher discontinuation rate is not necessarily at risk of unintended pregnancy due to the fact that desire to become pregnant is one of the most important reproductive rights of women..

The overall mean duration of LARCs use was four folds less than the recommended duration of use for the three LARCs analysed in this study. This finding could imply the risk of unintended pregnancy due to improper use of LARC methods. This finding has lower duration of use when compared to findings from other parts of Ethiopia [21, 22] and in UK [23] which ranged from 19.5 months to 27 months for implanon. In addition, the mean duration of LARC methods is lower than the findings from demographic health survey (DHS) of 14 developing countries where the mean duration of uninterrupted IUCD use was 37 months [24]. However, as to our knowledge there were no studies for Jadelle to compare mean duration of use. In any case of the above findings, our findings suggest that there might be the risk of improper use of LARC methods which in turn leads to unintended pregnancy. Therefore, the findings of study imply many efforts should be made to improve average duration of use of LARC methods to reduce the risk of unintended pregnancy.

Three in ten women in this study switched from the methods they were using to any other modern contraceptive methods. This is slightly higher than a study done in other parts of the country, Dilla (27.6%) [25]. Here, as long as all methods are effective in preventing unintended pregnancy, the women may prefer to another method following discontinuation. But, switching from LARC methods to modern short-acting family planning methods may widen risk of unintended pregnancy and switching from one type of LARC to another type may also lead to risk of pregnancy. In our study, a significant number of women switched from previously used specific LARC methods to short acting family planning that may also expose them for unintended pregnancy. Our finding is higher than the findings from Bangladeshi (15.4%) [13] and Senegal (17%) [26]. Whereas, the findings of this study is lower than the finding reported in Ethiopia [5–7, 13]. Switching from LARC methods to other short-acting methods (less effective methods) might be due to failure to counselling, providers’ gender factor, position and sector, provider myths that this finding call for implementing strategies to increase continuation rate of utilization of long acting family planning methods [6]. Moreover, higher proportion of Switching from one type of LARC methods to the other LARC methods might be another odd finding of this study, implying that the methods may have been improperly inserted. Hence, additional studies should be conducted regarding to competency of family planning providers so that their skill gap regarding insertion of LARC methods may be filled with additional training.

The two most cited reasons mentioned by the women for discontinuation of LARC methods in our study were desire to get pregnant and method specific side effect. Despite there is a difference in the design of the study, the findings of our study is consistent with findings extracted from the Ethiopian demographic health survey [22]. Similarly, reasons for implanon removal were similar with the study done in South Africa, which showed implanon was removed by the side effect and a desire for more pregnancies [27]. Moreover, side effect was also the main reason citedin the study done in Zambia (11%) [28] and consistent with the findings from the African population and health research centre for East African [29].

In our logistic regression analysis, only number of living children of women was significantly associated with discontinuation of long acting reversible family planning methods. Although the design of the study varies, our finding is consistent with study done in Ethiopia [22]. However, in our finding primiparous women were more likely to discontinue when compared to the grand multipara women whereas a demographic health analysis indicated that higher number of living children is associated with discontinuation [22]. While other variables like age of women, duration of use of LARCs, and educational status were not associated with discontinuation in our findings. Similarly, there was no single variable associated with switching of LARC methods.

### Limitation and strength of the study

Huge sample size of the study could be among the strengths of our study. In addition, the finding of our study may suggest poor quality of family planning services as is a facility based. Our study is a facility based that may result in higher estimation of discontinuation and switching off LARC methods than the survey design in the community due to high risk of removal of the method during revisit. This may unable to generalize to the general target groups in the community. Some of the most important variables that may have an association with discontinuation and switching off LARC methods are not available in the standard registration book. These variables could be confounders but we were unable to analyse.

## Conclusion and recommendation

Our finding showed that discontinuation of long acting reversible family planning method was high. Method switching from long acting reversible family planning methods to short acting family planning methods was also high in our finding that may expose the women to unintended pregnancy. Desire to get pregnant and method specific side effect were most common reasons for both method discontinuation and method switching. In our study, primiparous women (those women having one living children) were more likely to discontinue use of long acting reversible family planning methods. Hence, re-evaluating family planning services focusing on how can effectively counsel side effects of corresponding long acting reversible family planning methods and training should also be provided for community health workers. Further, context based implementation research should be required to design more effective methods of family planning information for women as well as their sexual mates.

## Supplementary Information

**Additional file 1.** Checklist for magnitude and factors for discontinuation and switching off long acting reversible contraceptive users in Hadiya Zone, Ethiopia, 2019.

## Data Availability

All data are available within the manuscript. The datasets used and/or analysed during the current study are available from the corresponding author based on reasonable request.

## Abbreviations

LARCs: Long acting reversible contraceptives
LAFP: Long acting family planning
WURTH: Wachemo University Referral Teaching Hospital
MDGs: Millennium development goals
IUCD: Intrauterine contraceptive device
EDHS: Ethiopian Demographic Health Survey
PH: Primary Hospital
HSTP: Health sector transformation plan
