# Supplementary material for: Magnitude and factors for method discontinuation and switching among long acting reversible contraceptive users in health facilities of Southern Ethiopia
Source: Reprod Health. 2022 Feb 19;19:47. doi: 10.1186/s12978-022-01357-2 (PMC8858462; doi:10.1186/s12978-022-01357-2)
Supplement: Supplementary file 1 — Additional file 1. Checklist for magnitude and factors for discontinuation and switching off long acting reversible contraceptive users in Hadiya Zone, Ethiopia, 2019. [file 12978_2022_1357_MOESM1_ESM.docx]

**Annex-1-Checklist for magnitude and factors for discontinuation and switching off long acting reversible family planning users in Hadiya zone, Ethiopia, June 2019.**

| ***Section 1. Identification: Personal information*** | | | |
| --- | --- | --- | --- |
| Code | | MRN |  |
| Q101 | | Age of the mother | ____________ years |
| Q102 | | Place of residence ( to be checked /collected from the women’s card) | 1. Rural 2. Urban |
| Q103 | | Marital status ( to be checked /collected from the women’s card) | 1. Single 2. Married 3. Divorced 4. Widowed |
| Q104 | | Educational status ( to be checked /collected from the women’s card) | 1. Illiterate 2. Primary education 3. Secondary education 4. College or above |
| Q105 | | The numbers of times a woman has been pregnant( to be checked /collected from the women’s card) | __________________ |
| Q106 | | The numbers of births a woman has been delivered at 24 weeks or more of gestational age ( to be checked /collected from the women’s card) | __________________ |
| ***Section 2. Family Planning Long acting Removal services:*** | | | |
|  | ***Registration*** | |  |
| Q201 | Reg. date  (DD/MM/YY) | | Day / Month / Year (DD/MM/YY)  ____/_____/_____ |
| Q202 | Date of insertion | | Day / Month / Year (DD/MM/YY)  ____/_____/_____ |
| Q203 | Type of LAFP used | | Write Contraceptive method provided (record modern methods only) write type of as contraceptive follows   1. Implanon **-**Implanon Implant 2. Sino-Implant-Sino Implant 3. Jadell -Jadelle Implant 4. IUD- Intrauterine Contraceptive Device |
| Q204 | Did the mother take her LAFP other than this facility previously | | 1. Yes  2. No |
| Q205 | If ye for Q105, the facility where the LAFP provided? | | 1. Hospital 2. Health center 3. Health post 4. Private clinics |
| Q106 | *Date of removal service provided* | | Day/Month/Year(DD/MM/YY  ____/_____/_____ |
| Q207 | *LAFP method duration used in month* | | ______ (Write duration of method used in month (if the client used only one month we can put =1, if it is two month =2 and so on ) |
| Q208 | *Reasons of removal* | | 1. On recommended time 2. Side effect 3. Want to get pregnant 4. Misconception 5. Others(Specify)______________ |
| ***Section 3. Counseling and testing*** | | | |
| Q301.1. | | HIV test offered | 1. Yes  2. No |
| Q301.2 | | HIV test performed | 1. Yes  2. No |
| Q301.3 | | HIV Test results (P/N) | 1. Yes  2. No |
| Q301.4 | | HIV specific contraceptive counseling offered | 1. Yes  2. No |
| Q301.5 | | Positive and linked to ART | 1. Yes  2. No |
| Q302 | | Targeted population category ( an individual needs to be assigned only in one category that best describe him/her. | 1. Female Commercial Sex workers 2. Long distance drivers 3. Mobile/Daily Laborers 4. Prisoners 5. OVC 6. Children of PLHIV 7. Partners of PLHI 8. Other MARPS 9. General population |
| Q303 | | Post removal contraceptive provided (Contraceptive method provided (Circle modern methods only)) | 1. MaC -Male Condom 2. FeC - Female Condom 3. OC - Oral Contraceptive 4. Inj - Injectable 5. EC - Emergency Contraception 6. Diaph - Diaphragm 7. IUCD- Intrauterine Contraceptive Device 8. Imp –Implant |
